# L-Citrulline Influences the Body Temperature, Heat Shock Response and Nitric Oxide Regeneration of Broilers Under Thermoneutral and Heat Stress Condition

**DOI:** 10.3389/fphys.2021.671691

**Published:** 2021-08-11

**Authors:** Victoria A. Uyanga, Minghui Wang, Tian Tong, Jingpeng Zhao, Xiaojuan Wang, Hongchao Jiao, Okanlawon M. Onagbesan, Hai Lin

**Affiliations:** ^1^Department of Animal Science, College of Animal Science and Veterinary Medicine, Shandong Provincial Key Laboratory of Animal Biotechnology and Disease Control, Shandong Agricultural University, Tai’an, China; ^2^Department of Animal Physiology, Federal University of Agriculture, Abeokuta, Nigeria

**Keywords:** amino acid, L-citrulline, heat stress, inflammation, broilers

## Abstract

Heat stress (HS) adversely affects several physiological responses in organisms, but the underlying molecular mechanisms involved are yet to be fully understood. L-Citrulline (L-Cit) is a nutraceutical amino acid that is gaining research interest for its role in body temperature regulation and nitric oxide synthesis. This study investigated whether dietary supplementation with L-Cit (1% of basal diet) could ameliorate the effects of acute HS on thermotolerance, redox balance, and inflammatory responses of broilers. Ross 308 broilers (288 chicks) were subjected to two environments; thermoneutral at 24°C (TNZ) or HS at 35°C for 5 h, and fed two diets; control or L-Cit. The results showed that HS increased the ear, rectal (RT), and core body (CBT) temperatures of broilers, along with higher respiratory rate. The RT and CBT readings were intermittently affected with time effect, whereas, L-Cit supplementation lowered the mean CBT than the control diet. Antioxidant assays showed that superoxide dismutase was increased during HS, while, catalase was promoted by L-Cit supplementation. In addition, L-Cit induced glutathione peroxidase activity compared to the control diet during HS. Hypothalamic heat shock protein (HSP)-90 was upregulated by HS, but L-Cit downregulated heat shock factor (HSF)-1, and HSP 60 mRNA expressions. HSF 3 mRNA expression was downregulated by L-Cit under TNZ condition. More so, HS increased the plasma nitric oxide (NO) concentration but lowered the total NO synthase (tNOS) activity. In contrast, L-Cit supplementation limited NO production but increased the tNOS activity. Arginase activity was increased in the control fed group during HS but L-Cit supplementation lowered this effect. The NOS-COX pathway was significantly affected under TNZ condition, since L-Cit supplementation downregulated the mRNA expression of iNOS-COX2 in the hypothalamus, and further reduced the serum PGE2 concentration. Together, these data indicates that L-Cit influenced the antioxidant defense, heat shock response and nitric oxide regeneration both under thermoneutral and HS conditions; and that L-Cit may be directly and/or indirectly involved in the central regulation of body temperature.

## Introduction

Heat stress (HS) adversely impacts poultry production by altering several neuroendocrine, molecular, and physiological processes, which are still not completely understood. It poses deleterious effects on the health, production, and welfare of humans and livestock, as such demanding the persistent search for novel and effective strategies that would mitigate its multifaceted impacts on productivity and sustainability. HS influences several physiological responses in the body, and direct assessment of metrics such as rectal temperature (RT), surface temperature, respiratory rate, panting, and heat production, can be used to examine the responsiveness and adjustment during acute or chronic HS (Yahav, 2000; Renaudeau et al., 2012). Transcriptional analysis has shown that HS strongly over-represents several pathways such as the electron transport chain, apoptosis, heat shock response, inflammatory response, and oxidative stress signaling (Kapila et al., 2016). Identification of these transcriptional responses would assist in understanding the responsiveness of organisms to environmental heat load.

Broilers subjected to HS often undergo physiological responses, such as hyperthermia, metabolic disorders, oxidative stress, respiratory alkalosis, systemic inflammation, and mortality (Loyau et al., 2014; Brownstein, 2016). Moreover, thermal and oxidative stress initiates the expression and chaperoning activity of inducible heat shock proteins, which function to protect cells from damage (Zhao et al., 2006). An important physiological response during HS is an increase in blood flow to the body surface or upper respiratory tract (Majdeddin et al., 2020). These perturbations are necessary to provide the fluid needed for heat dissipation during panting which is accompanied by peripheral vasodilation and an increase in body temperature (Yahav et al., 1997). Nitric oxide (NO) is an active effector of vasodilation that directly regulates vascular tone and blood flow in the vascular smooth muscle (Luiking et al., 2010). The primary precursor for the endogenous synthesis of NO by nitric oxide synthase (NOS) is L-arginine. Therefore, increasing arginine availability via L-arginine to L-citrulline (L-Cit) recycling might be beneficial for heat-stressed birds.

L-Citrulline, a non-protein amino acid found mainly in watermelon, is an important metabolite of the urea cycle (Azizi et al., 2020). Recently, L-Cit has been revealed as a nutritional supplement with the ability to lower body temperature and it can afford thermotolerance during thermal challenge in chicks (Chowdhury et al., 2021). It was demonstrated that a single oral dose of L-Cit (15 mmol/10 ml/kg body weight) could induce hypothermia in chicks throughout the 5 h study period, whereas, dual administration of L-Cit was necessary to sustain thermotolerance in chicks (Chowdhury et al., 2017). L-Cit is regarded as a HS biomarker since changes in the plasma L-Cit concentration can reflect the extent and length of thermal challenge in chicks (Chowdhury et al., 2014; Chowdhury, 2019). Plasma L-Cit level is increased during short periods of HS, but exposure to long term HS reduces circulating L-Cit concentration (Chowdhury et al., 2014; Chowdhury, 2019). Kvidera et al. (2016) reported that L-Cit slightly affected thermal response during cyclical HS in gilts by decreasing respiratory rate, with a tendency to decrease the RT. In another study, dietary supplementation of 1% L-Cit decreased the respiration rate during HS but did not influence the RT (Liu et al., 2019). These researches demonstrate the role of L-Cit in thermoregulation, however, there is still paucity of information regarding the molecular responses and mechanisms responsible for these actions. Importantly, L-Cit can be recycled during L-arginine generation and NO synthesis (Bahri et al., 2013). The three NOS isoforms; neuronal NOS (nNOS), inducible NOS (iNOS), and endothelial NOS (eNOS) can catalyze L-arginine, to generate NO and L-Cit (Schwedhelm et al., 2008). Dietary L-Cit supplementation increased the serum amino acid concentration for arginine, citrulline, and ornithine in laying hens (Uyanga et al., 2020). Studies have shown that L-Cit can effectively increase systemic arginine levels (Jegatheesan et al., 2015; Kim et al., 2015), much higher than direct L-arginine supplementation (Lassala et al., 2009; Morita et al., 2014; Agarwal et al., 2017). L-Arginine, an essential amino acid in poultry nutrition, mediates several biological functions during stress conditions (Wideman et al., 1995; Fouad et al., 2012; Varasteh et al., 2018). However, L-arginine deficiency may occur due to increased catabolism, L-Cit unavailability for *de novo* synthesis, and under certain inflammatory conditions (LeBaron et al., 2020). Thus, augmenting L-arginine availability would provide a measure to promote NO synthesis and bioavailability during stress condition. In addition, the application of L-Cit in animal production is yet an avenue to explore for its direct benefits, as well as its indirect actions via arginine and NO generation (Uyanga et al., 2020).

Nitric oxide is involved in a wide range of physiological functions, including anti-oxidative (Hayashi et al., 2005), anti-inflammatory, appetite regulation (Zendehdel et al., 2017), vasodilation (Flam et al., 2007), and thermoregulation (Coleone et al., 2009; Dantonio et al., 2016). NO is an important modulator of body temperature because it can act on the central nervous system (CNS) and in the periphery to elicit thermoregulatory responses (Simon, 1998; Steiner and Branco, 2001). NO decreases body temperature at sites of thermo-integration, whereas, it acts to increase body temperature at pyrogenic and thermo-perceptive regions within the CNS (Gerstberger, 1999). The hypothalamus functions as the thermoregulatory center, where NO secondary target, soluble guanylate cyclase is abundant (Gerstberger, 1999). Stress-induced hyperthermia and infection-induced fever are two mechanisms that lead to an increase in body temperature via activation of the autonomic nervous system and the immune system respectively (Briese and Cabanac, 1991; Oka et al., 2001; Vinkers et al., 2009). The rise in body temperature induced during stress, is considered relatively similar to fever caused by exogenous pyrogens (such as lipopolysaccharides) in animals (Soszynski, 2001; Vinkers et al., 2009). Stress-induced fever is associated with the upregulation of pro-inflammatory cytokines, suggesting that cytokines play an important role in body temperature regulation (Evans et al., 2015). Pyrogenic cytokines, including interleukin-1β (IL-1β), interleukin-6 (IL-6), and tumor necrosis factor-α (TNF-α), can induce fever when released peripherally or centrally within the CNS (Monroy et al., 2001). The activity of these cytokines requires activation of the cyclooxygenase (COX) pathway, for the subsequent release of prostaglandin E2 (PGE2) (Lazarus, 2006; Krall et al., 2010). PGE2 is recognized as a final mediator of fever and is responsible for the upward resetting of the thermoregulatory set point in the preoptic area of the anterior hypothalamus (PO/AH) (Lazarus, 2006; Evans et al., 2015). Since high temperature results in immune dysfunction, inflammation, and in extreme cases, multiple organ disorders (Ahmed, 2005), we investigated the involvement of COX-PG signaling during HS condition. Therefore, this study examined the effects of L-Cit supplementation on thermotolerance, redox balance, heat shock response, nitric oxide regeneration, and inflammatory responses of acute heat-stressed broilers.

## Materials and Methods

### Experimental Animals

All experimental procedures were conducted in compliance with the “Guidelines for Experimental Animals” of the Ministry of Science and Technology (Beijing, China), and the study was approved by the Ethics Committee of Shandong Agricultural University, China.

One-day-old Ross 308 chicks (288 birds) were randomly assigned to four environmentally controlled chambers for rearing. Each chamber had three (3), double-tier battery cage units, with four cages per tier (cage dimension: 47.5 × 37 × 36 cm), and each cage housed three birds. Chicks were fed either a basal diet (corn-soy-based diet, Control) or a basal diet supplemented with 1% L-Cit to meet the National Research Council (NRC) recommendation (Table 1). L-Cit was purchased from Shandong Fosun Biotechnology Co., Ltd., China, and the dosage was adapted from a previous study (Uyanga et al., 2020). The ambient temperature was adjusted gradually from 32°C, 60% RH from d 1 until it reached 24°C, 60% RH on d 21. From d 21, the birds were fed finishing diets, and on d 24, six birds per treatment were implanted with a thermochron temperature logger within the abdominal cavity for continuous recording of core body temperature (CBT). A midline laparotomy was performed, and a temperature-measuring device was inserted into the peritoneal cavity. The abdominal muscles and skin were sutured, and the birds were allowed to recover for 3 days (Ben-Hamo et al., 2010).

**TABLE 1:** Composition and nutrient levels of basal diets (as-fed basis) %.

Ingredients	Starter		Finisher	
	Control	L-Citrulline	Control	L-Citrulline
Corn (8.5% CP)	54.99	60.22	59.68	64.81
Soybean meal (43% CP)	37.02	31.23	31.65	25.94
Soybean oil	3.87	2.94	4.72	3.80
Limestone	1.19	1.20	1.27	1.28
CaHPO_4_	1.68	1.76	1.63	1.70
NaCl	0.30	0.30	0.28	0.29
L-Citrulline	–	1.00	–	1.00
L-Lys⋅HCl (99%)	0.21	0.34	0.18	0.31
DL-Met (99%)	0.21	0.23	0.14	0.17
L-Thr (99%)	–	0.09	–	0.09
L-Arg (99%)	0.02	0.18	–	0.16
Choline chloride (50%)	0.26	0.26	0.20	0.20
Vitamin premix^1^	0.05	0.05	0.05	0.05
Mineral premix^2^	0.20	0.20	0.20	0.20
Total	100.00	100.00	100.00	100.00
Nutrient levels				
CP	21.0	21.0	19.0	19.0
ME/(MJ/kg)	12.55	12.55	12.97	12.97
Ca	0.90	0.90	0.90	0.90
Non-phytate P	0.45	0.45	0.43	0.43
Lys	1.242	1.242	1.095	1.095
Met	0.537	0.537	0.449	0.449
Met + Cys	0.889	0.861	0.775	0.747
Thr	0.861	0.861	0.774	0.774
Arg	1.389	1.389	1.220	1.220

*^1^Vitamin premix provided the following per kilogram of diet: VA (retinyl acetate) 10,000 IU, VD_3_ (cholecalciferol) 2,000 IU, VE (DL-α-tocopheryl acetate) 11.0 IU, VK 1.0 mg, VB_1_ 1.2 mg, VB_2_ 5.8 mg, VB_6_ 2.6 mg, VB_12_ 0.012 mg, niacin 66.0 mg, pantothenic acid (calcium pantothenate) 10.0 mg, biotin 0.20 mg, folic acid 0.70 mg.*

*^2^Mineral premix provided the following per kilogram of diet: Mg 100 mg, Zn 75 mg, Fe 80 mg, I 0.65 mg, Cu 8.0 mg, Se 0.35 mg.*

On d 28, birds were conditioned into two environmental temperatures; thermoneutral at 24°C (TNZ) and acute HS at 35°C for 5 h. Of the four environmental chambers used, two chambers were assigned to either HS or TNZ condition. The birds were arranged such that each treatment was evenly represented in the two chambers used (i.e., 4 replicates × 9 chickens for TNZ + Control, and 4 replicates × 9 chickens for TNZ + L-Cit were housed together). This was performed to minimize variations in the environmental housing. The feed was withdrawn overnight before the commencement of the experiment to minimize metabolic heat load and heat production (Vinales et al., 2019). Drinking water was provided *ad libitum* during the study period.

### Experimental Procedures and Samples Collection

Each trial commenced after the environmental chambers had reached the target temperature designed for the experiment (∼1 h). The temperature and relative humidity of the chambers were monitored continuously throughout the experiment. Blood was collected from the wing vein of individual birds into heparinized and non-heparinized tubes, and centrifuged at 3,000 × *g* for 10 min at 4°C to obtain plasma and serum samples respectively (Jiang et al., 2020; Guo et al., 2021). Samples were stored at −20°C until analysis. Chickens were sacrificed by decapitation and exsanguination after 5 h HS, and tissue samples were excised, weighed, snap-frozen in liquid nitrogen, and stored at −80°C for further studies. The hypothalamus was dissected from the brain using mini tweezers as previously described (Piorkowska et al., 2018). Relative organ weight was computed as organ weight/body weight of chicken x 100.

### Body Temperature Measurement

The RT and CBT were measured during the course of the experiment, whereas the ear temperature and respiratory rate of chickens were measured at the end of the 5 h HS treatment. Ear temperature was measured using an infrared thermal imager (TP 160 – Suzhou Shengguang Instrument Co., Ltd., China). This technique has been reported to provide precision and accuracy (Yahav et al., 2005; Yahav and Giloh, 2012). RT was detected using KRUUSE Vet thermometer (Cat No. 291110, China), by inserting the probe at a depth of 2–3 cm through the cloaca into the rectum (Uyanga et al., 2020).

The respiratory rate was recorded with a video camera and computed as the number of breaths per minute per bird. Two birds per replicate were recorded to capture the inspiration, and expiration cycle during respiration for 3–5 min, and the recordings were automatically saved for later viewing. From the recorded videos, the number of breaths per bird within 60 s was obtained and used to compute the respiratory rate. CBT was measured using a thermochron logger (iButton, DS1922L, Maxim, CA, United States) at 30 min intervals, and plotted over 300 min. Data were obtained using the One wire Viewer software (Maxim Integrated Products Inc., CA, United States).

### Plasma Metabolites and Hormone Determination

Plasma circulating metabolites including glucose (GLU), total protein (TP), and uric acid (UA) levels were measured spectrophotometrically using a Hitachi L-7020 automatic biochemical analyzer (Hitachi High-Technologies Corp., Tokyo, Japan). Plasma triglyceride (TG) and total cholesterol (TCHO) levels were measured using assay kits according to the manufacturer’s instructions (Jiancheng Bioengineering Institute, Nanjing, Jiangsu, China). The absorbance was determined at 540nm (Elx808, Bio-Tek Winooski, VT, United States).

The DetectX^®^ Corticosterone Enzyme Immunoassay Kit (Arbor Assays, MI, United States) was used to measure corticosterone concentration. Samples were pipetted into antibody-coated microtiter plates, and a corticosterone-peroxidase conjugate was added. The addition of a polyclonal antibody initiates a binding reaction to corticosterone. Subsequently, 3,3′,5,5′-Tetramethylbenzidine (TMB) substrate solution was added to react with the bound corticosterone-peroxidase conjugate. The reaction was read at 450nm using a microplate reader (Elx808, Bio-Tek, Winooski, VT, United States). The assay parameters were intra-assay CV < 10%, and inter-assay CV < 10%

A chicken 3′-triiodothyronine ELISA Kit (Shanghai MLBIO Biotechnology Co., Ltd., China) was used to measure the thyroid hormone (T_3_) concentration. The assay used purified chicken T_3_ antibody-coated microtiter plate wells, to form solid-phase antibodies. The detected antibodies were labeled with horseradish peroxidase (HRP) to form an antibody-antigen-enzyme-antibody complex, which gave a blue colored reaction with the addition of TMB substrate solution. The reaction was terminated by the addition of sulfuric acid solution and the color change was measured spectrophotometrically (Elx808, Bio-Tek, Winooski, VT, United States) at a wavelength of 450 nm. The concentration of each sample was then determined by comparing the optical density of the sample to the standard curve.

As described for T_3_, a similar procedure was performed for the detection of PGD2, PGE2, and Arginase using Chicken Prostaglandin D2 (PGD2) ELISA Kit, Chicken Prostaglandin E2 (PGE2) ELISA Kit, and Chicken Arginase-1 (ARG) ELISA kit respectively (Shanghai MLBIO Biotechnology Co., Ltd., China). The sample concentration was measured using a microplate reader (Elx808, Bio-Tek, Winooski, VT, United States) at a wavelength of 450 nm. The assay parameters were intra-assay CV < 10%, and inter-assay CV < 15%. A standard curve and linear regression equation was generated to determine the test concentration.

### NO Concentration and NOS Activity

The NO concentration was measured using a commercial kit (Jiancheng Bioengineering Institute, Nanjing, Jiangsu, China), as previously reported (Wang et al., 2018; Uyanga et al., 2020). NO is a free radical, that can rapidly oxidize to NO_2_^–^ in the blood. In this reaction, nitrate reductase catalyzes the reduction of nitrate to nitrite, and the sum of nitrate and nitrite concentrations (NO_2_^–^ + NO_3_^–^) is used to represent the total nitric oxide concentration *in vivo* (Bowen et al., 2007). The reaction absorbance was read using 0.5cm cuvettes at 550 nm wavelength, with a spectrophotometer (Beijing PGeneral, Beijing, China).

Nitric oxide synthase activity was measured for total NOS (tNOS), iNOS, and eNOS, using commercial kits according to the manufacturer’s protocols (Jiancheng Bioengineering Institute, Nanjing, Jiangsu, China). The NOS activity assay is an enzymatic process that is based on the biochemical conversion of arginine and molecular oxygen to produce citrulline and NO. The NO formed reacts with nucleophilic substances to produce non-ferrous compounds. The reaction occurs with co-factors including reduced nicotinamide adenine dinucleotide phosphate, oxygen, calcium, calmodulin, and tetrahydrobiopterin. The presence or absence of calcium was used to determine the calcium-dependent activity of constitutive NOS (tNOS) and the calcium-independent activity of iNOS. The absorbance of tNOS and iNOS were measured using 1 cm cuvettes at 530 nm using a UV-2450 spectrophotometer (Beijing PGeneral, Beijing, China). The assay for eNOS was performed in a 96-well plate using a microplate reader (Elx808, Bio-Tek, Winooski, VT, United States) at a wavelength of 450 nm. Assays parameters were an intra-assay CV < 10%, inter-assay CV < 12% and, sensitivity range 0.05 to 20 ng/mL. A standard curve and linear regression equation were generated using ELISAcalc software to determine chicken eNOS activity.

### Detection of Oxidative Damage

The antioxidant defense capacity including total-antioxidant capacity (T-AOC), catalase (CAT), superoxide dismutase (SOD), glutathione peroxidase (GSH-Px), and the malondialdehyde (MDA) content in the plasma were determined using commercial kits according to the manufacturer’s instructions (Jiancheng Bioengineering Institute, Nanjing, Jiangsu, China).

T-AOC was measured using the ferric reducing antioxidant power assay (FRAP) principle. Under acidic conditions, antioxidants can react with ferric tripyridyltriazine (Fe^3+^-TPTZ) complex and convert it into ferrous tripyridyltriazine (Fe^2+^-TPTZ). The absorbance was measured at 570 nm. SOD activity was measured using the WST-1 (2-(4-Iodophenyl)-3-(4-nitrophenyl)-5-(2,4-disulfophenyl)-2H-tetrazolium, monosodium salt) method. In this assay principle, xanthine oxidase catalyzes the reduction of WST-1 by superoxide anion, to generate a water-soluble formazan dye. The SOD activity was quantified with an intra-assay CV of 5.05%, inter-assay CV of 3.32%, and absorbance at 450nm. CAT activity was determined using the visible light method. The assay is based on the measurement of hydrogen peroxide (H_2_O_2_) substrate after it is decomposed by the catalase enzyme. The remaining H_2_O_2_ reacts with ammonium molybdate to form a complex which was determined at 405 nm and calculated as CAT activity. The GSH-Px enzyme catalyzes the reaction of H_2_O_2_ and reduced glutathione to produce H_2_O and oxidized glutathione. The GSH-Px activity was expressed by measuring the consumption of reduced glutathione at 412 nm. In addition, the MDA assay protocol was based on the thiobarbituric acid (TBA) principle. The reaction generates an MDA-TBA product which was quantified at 540 nm. The reaction absorbance for T-AOC, SOD, CAT, GSH-Px, and MDA were measured using a microplate reader (Elx808, Bio-Tek Winooski, VT, United States).

### RNA Extraction and Quantitative Real-Time PCR Analysis

Total RNA from hypothalamic tissue was extracted by the acid phenol method using NcmZol reagent (NCM Biotech, China), following the manufacturer’s instructions. RNA concentration and purity were detected using the nucleic acid spectrophotometer (DeNovix DS-11, United States) with absorbance values at 260nm and 280 nm (A260/280 = 1.80 − 2.01). RNA was reverse-transcribed to complementary DNA (cDNA) using PrimeScript^TM^ reverse transcription reagent kit with gDNA eraser (Takara Bio Inc., Japan) according to manufacturer’s protocol. Real-time PCR was performed on ABI QuantStudio 5 Real-Time PCR Instrument (Applied Biosystems, Thermo Fisher Scientific, United States) using 2 μL of 5x diluted cDNA template, 0.8 μL of each forward and reverse primer, 10 μL of TB Green^TM^ Premix ExTaq^TM^ (Takara Bio Inc., Japan), 0.4 μL of ROX reference Dye II and 6 μL DEPC water, in a total of 20 μL reaction mix. Primers used for qRT-PCR were designed by Beacon Designer 8 software and were based on published target sequences (Table 2). Primers were normalized against the mRNA level of β-actin as an internal control and the control diet group under thermoneutral condition was used as calibrator. Thermal cycling was initiated with an initial denaturation stage of 30 s at 95°C, and this stage was followed by 40 cycles of 95°C for 5 s and 60°C for 30 s. The relative expression of the target genes was analyzed using the 2^–ΔΔCT^ method.

**TABLE 2:** List of primers used for real-time PCR analysis.

Gene	Primer sequence (5′ to 3′)	Orientation	^*a*^Accession No.
nNOS	CTCGGATGCACGGAAGTCCT	Forward	XM_004934480.1
	CGTGAACCCAGCCCAAACAC	Reverse	
iNOS	GTGGTATGCTCTGCCTGCTGTTG	Forward	NM_204961
	GTCTCGCACTCCAATCTC TGTTCC	Reverse	
eNOS	GGATGTGCTGCACGGTCTGC	Forward	JQ434761.1
	AGGACGTGCTGCGGACACAG	Reverse	
COX-2	GCACCGTTCTCCCTGAAAGG	Forward	NM_001167718.1
	GTTGCCTCTGTGGGTTCAGG	Reverse	
HSF 1	CAGGGAAGCAGTTGGTTCA CTACACG	Forward	L06098.1
	CCTTGGGTTTGGGTTG CTCAGTC	Reverse	
HSF 3	TCCACCTCTCCTCTCGGAAG	Forward	L06126.1
	CAACAGGACTGAGGAGCAGG	Reverse	
HSP 60	AGAAGAAGGACAGAGTTACC	Forward	NM_001012916.1
	GCGTCTAATGCTGGAATG	Reverse	
HSP 70	TCTCATCAAGCGTAACACCAC	Forward	JX827254.1
	TCTCACCTTCATACACCTGGAC	Reverse	
HSP 90	ATGCCGGAAGCTGTGCAAA CACAGGACCAA	Forward	NM_001109785.1
	GGAATCAGGTTAATTTT CAGGTCTTTTCCA	Reverse	
IL-6	CTCCTCGCCAATCTGAAGTC	Forward	HM179640.1
	AGGCACTGAAACTCCTGGTC	Reverse	
TNF-α	GAGCGTTGACTTGGCTGTC	Forward	HQ739087.1
	AAGCAACAACCAGCTATGCAC	Reverse	
β-Actin	TGCGTGACATCAAGGAGAAG	Forward	NM_205518
	TGCCAGGGTACATTGTGGTA	Reverse	

*^*a*^Accession number refers to Gene bank (NCBI). nNOS, neuronal NOS; iNOS, inducible NOS; eNOS, endothelial NOS; COX-2, cyclooxygenase 2; HSF1, heat shock factor 1; HSF3, heat shock factor 3; HSP60, heat shock protein 60; HSP70, heat shock protein 70; HSP 90, heat shock protein 90; IL-6, interleukin-6, TNF-α, tumor necrosis factor-alpha.*

### Statistical Analysis

Data were analyzed using two-way ANOVA with Environment (TNZ vs. HS) and Diet (Control vs. L-Cit) as the main effects, using Statistical Analysis Software (SAS version 8.1; SAS Institute Inc., Cary, NC, United States). RT and CBT data were analyzed using three-way repeated measures ANOVA to evaluate the main effects of environment, diet and time as well as their interactions. Duncan’s Multiple Range Test was used to analyze mean comparisons when the treatment effect was significant and presented as the means ± SEM. Charts were designed using GraphPad Prism, version 8.0.2 (GraphPad Software Inc., La Jolla, CA, United States). Differences were considered significant at *P* < 0.05

## Results

### HS and L-Cit Influences the Body Temperature of Broilers

**FIGURE 1:**
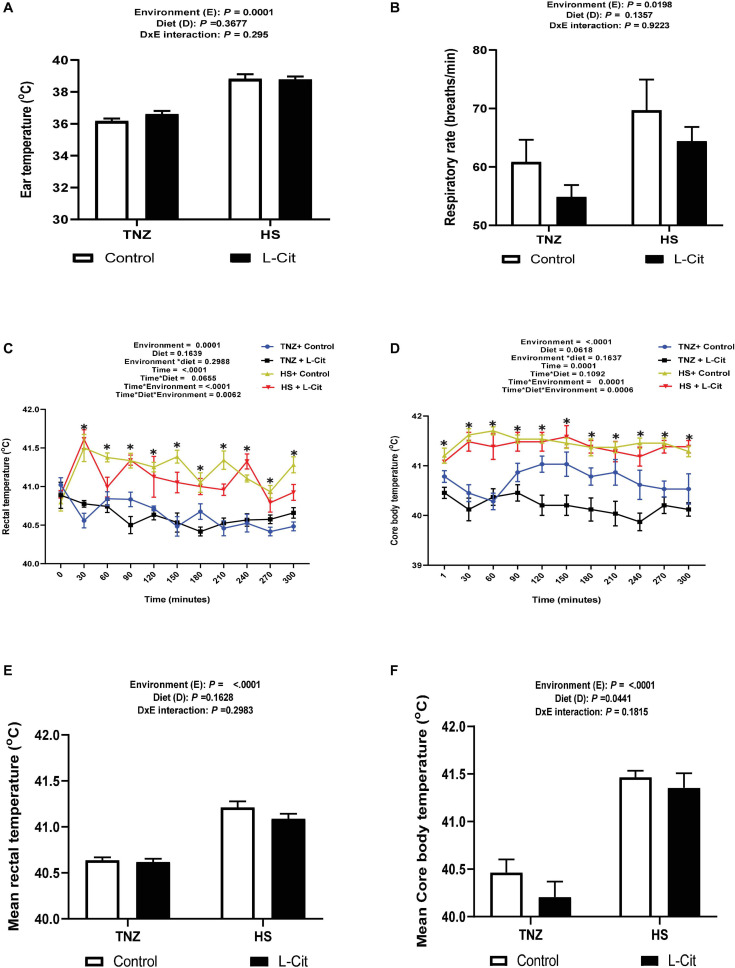
Effect of L-citrulline supplementation on thermotolerance of broilers during acute heat stress. **(A)** Ear temperature. **(B)** Respiratory rate. **(C)** Rectal temperature. **(D**) Core body temperature. **(E)** Mean rectal temperature. **(F)** Mean core body temperature Data are presented as mean ± SEM (*n* = 12). TNZ, thermoneutral; HS, heat stress. *Indicates significant effect of treatments at *P* < 0.05.

The effects of HS and L-Cit on the surface (ear) temperature, RT and CBT of broilers are presented in Figure 1. The ear temperature of broilers under HS condition was significantly higher than TNZ birds (*P* < 0.05, Figure 1A). Similarly, HS birds had an increased respiratory rate compared to the TNZ group (*P* < 0.05, Figure 1B).

Heat stress significantly increased the RT of broilers from the 30th min and lasted until the 300th min of study (Figure 1C). At the 60th min, HS + control fed broilers had the highest RT compared to the TNZ + control, TNZ + L-Cit and HS + L-Cit groups, which were statistically similar. At the 90th min, the HS groups fed both control and L-Cit diet had highest RT, followed by TNZ + control, while the TNZ + L-Cit broilers had the lowest RT. Also, at the 210th and 300th min, it was observed that HS + control broilers had the highest RT, followed by the HS + L-Cit group, while the TNZ groups fed either control or L-Cit had the lowest RT. Summarily, L-Cit supplementation was observed to lower RT at the 60, 210 and 300th min during HS, and at the 90th min under TNZ condition. At other time points, the HS groups fed both control and L-Cit were significantly higher than the TNZ groups fed either control or L-Cit.

The CBT was increased by HS exposure throughout the study (Figure 1D). At the 120th, 150th and 210th min, it was observed that TNZ + L-Cit broilers had lowered CBT than the TNZ + control, HS + control and HS + L-Cit groups, which were statistically similar. In addition, the CBT of the TNZ + control group was significantly higher than the TNZ + L-Cit group at the 180th and 240th min, but it was lower compared to the HS groups fed either control or L-Cit diets. In addition, the mean RT and CBT were elevated by HS compared to the TNZ group (*P* < 0.05; Figures 1E,F), although, L-Cit supplementation significantly decreased the mean CBT compared to the control diet (*P* < 0.05; Figure 1F).

### Acute HS and L-Cit Diet Affects Circulating Metabolites, Thyroid Hormone Levels, and Spleen Weight of Broilers

Table 3 shows that several organ indexes of broilers (heart, bursa, thymus, liver, and kidney) were not affected by the environment or diets. However, the spleen index was influenced (*P* < 0.05) by diet effect. Compared with the control diet, L-Cit supplementation significantly lowered the spleen index (*P* < 0.05) of broilers. Table 4 shows that the circulating plasma levels of TP, UA and TCHO were not significantly affected by environment or diet effects (*P* > 0.05). HS significantly reduced the GLU levels compared to the TNZ condition (*P* < 0.05). Furthermore, there was a significant interaction between environment and diet on the plasma GLU level. Broilers fed L-Cit at TNZ condition had higher GLU levels than those fed control diet at TNZ, or the HS birds fed either control or L-Cit diets (*P* < 0.05).

**TABLE 3:** Effect of heat stress and L-citrulline supplementation on organ index of broiler chicken.

Experimental groups	Final BW (g)	Heart (%)	Bursa (%)	Thymus (%)	Liver (%)	Kidney (%)	Spleen (%)
TNZ + control	444.54 ± 9.92	0.66 ± 0.02	0.35 ± 0.04	0.45 ± 0.03	2.60 ± 0.06	0.56 ± 0.05	0.19 ± 0.02
TNZ + L-Cit	455.04 ± 16.54	0.61 ± 0.03	0.29 ± 0.04	0.49 ± 0.04	2.80 ± 0.10	0.48 ± 0.04	0.12 ± 0.02
HS + control	451.29 ± 21.78	0.67 ± 0.02	0.35 ± 0.04	0.39 ± 0.03	2.79 ± 0.07	0.52 ± 0.05	0.17 ± 0.02
HS + L-Cit	451.50 ± 10.77	0.66 ± 0.02	0.39 ± 0.03	0.46 ± 0.03	2.71 ± 0.07	0.57 ± 0.05	0.14 ± 0.01
**Main effect**							
***Environment***							
TNZ	449.79	0.63	0.32	0.47	2.70	0.52	0.15
HS	451.39	0.66	0.37	0.43	2.75	0.55	0.15
***Diets***							
Control	447.91	0.66	0.35	0.42	2.70	0.54	0.17
L-Cit	453.27	0.63	0.34	0.48	2.76	0.52	0.13
***P*-Value**							
Environment	0.918	0.242	0.852	0.217	0.426	0.763	0.844
Diet	0.732	0.237	0.174	0.084	0.524	0.524	0.011
Interaction	0.743	0.389	0.178	0.755	0.069	0.187	0.347

*TNZ + control, thermoneutral + control diet; TNZ + L-Cit, thermoneutral + 1% L-citrulline diet; HS + control, heat stress + control diet; HS + L-Cit, heat stress + 1% L-citrulline diet; BW, body weight.*

**TABLE 4:** Effect of heat stress and L-citrulline supplementation on plasma metabolites of broiler chickens.

Experimental groups	TP (g/L)	UA (μmol/L)	GLU (mmol/L)	TG (mmol/L)	TCHO (mmol/L)
TNZ + control	26.16 ± 1.60	138.00 ± 25.04	11.05 ± 0.29^*b*^	1.59 ± 0.07^*a*^	2.99 ± 0.15
TNZ + L-Cit	27.84 ± 0.93	124.50 ± 20.91	12.37 ± 0.24^*a*^	1.35 ± 0.04^*b*^	3.60 ± 0.31
HS + control	26.99 ± 1.37	139.13 ± 18.61	10.86 ± 0.44^*b*^	1.37 ± 0.05^*b*^	3.09 ± 0.20
HS + L-Cit	25.18 ± 1.50	120.38 ± 10.17	10.56 ± 0.38^*b*^	1.35 ± 0.04^*b*^	3.31 ± 0.18
**Main effect**					
***Environment***					
TNZ	27.00	131.25	11.71	1.47	3.29
HS	26.08	129.75	10.71	1.36	3.42
***Diets***					
Control	26.58	138.56	11.00	1.48	3.04
L-Cit	26.51	122.44	11.47	1.35	3.68
***P*-Value**					
Environment	0.510	0.939	0.008	0.043	0.689
Diet	0.961	0.414	0.149	0.017	0.059
Interaction	0.215	0.894	0.026	0.036	0.374

*^*a,b*^Different letters indicate significant difference for interaction at *P* < 0.05. TNZ + control, thermoneutral + control diet;*

*TNZ + L-Cit, thermoneutral + 1% L-citrulline diet; HS + control, heat stress + control diet; HS + L-Cit, heat stress + 1% L-citrulline diet; TP, total protein; UA, uric acid; GLU, glucose; TG, triglyceride; TCHO, total cholesterol.*

**FIGURE 2:**
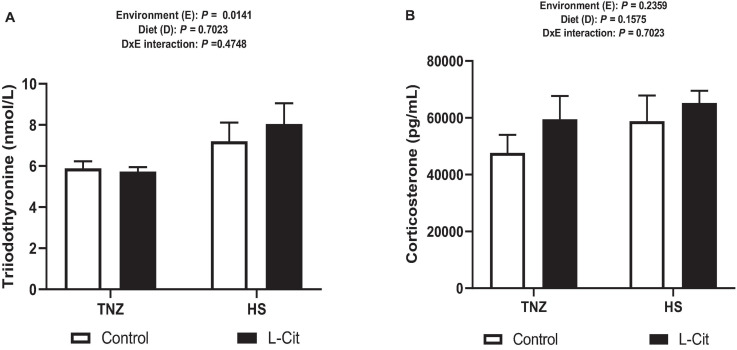
Effect of heat stress and L-citrulline supplementation on circulating hormone levels. **(A)** Triiodothyronine (T_3_). **(B)** Corticosterone levels. Data are presented as mean ± SEM (*n* = 8). TNZ, thermoneutral; HS, heat stress.

Heat stress significantly diminished the plasma TG content (*P* < 0.05) compared to birds under TNZ condition. L-Cit supplementation significantly decreased the TG content compared to the control fed group (*P* < 0.05). In addition, plasma TG was affected by a significant interaction between environment and diet (*P* < 0.05). It was observed that the TG content was highest for the control fed chickens than the L-Cit fed group at TNZ and the HS groups fed either control or L-Cit diets. Furthermore, HS significantly increased (*P* < 0.05) the levels of serum T_3_ hormone compared with TNZ condition (Figure 2A), but the serum corticosterone level was unchanged (Figure 2B; *P* > 0.05).

### L-Cit Supplementation Modulates the Antioxidant Status of Broilers

**FIGURE 3:**
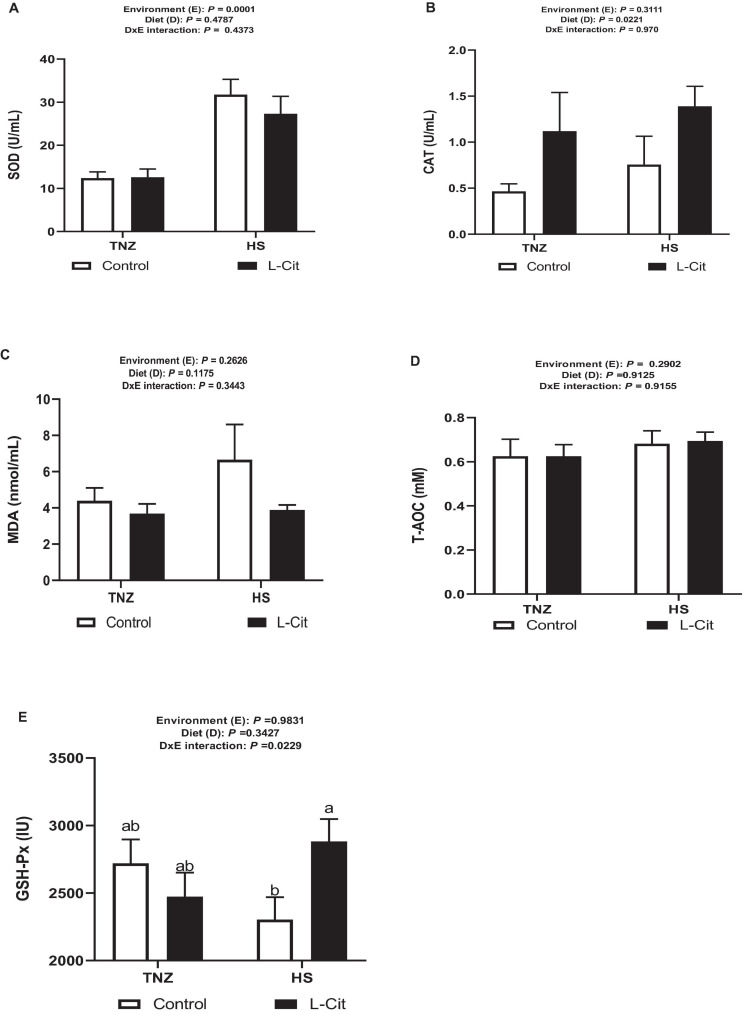
L-Citrulline supplementation modulates the anti-oxidant properties of broilers during acute heat stress. **(A)** Superoxide dismutase (SOD) activity. **(B)** Catalase (CAT) activity. **(C)** Malondialdehyde (MDA) content. **(D)** Glutathione peroxidase (GSH-Px) activity. **(E)** Total anti-oxidant capacity (T-AOC). Data are presented as mean ± SEM (*n* = 8). TNZ, thermoneutral; HS, heat stress. ^*a,b*^Different letters indicate significant difference of D×E interaction at *P* < 0.05.

As shown in Figure 3A, HS significantly increased the SOD activity (*P* < 0.05) compared to the TNZ group. CAT activity was increased by L-Cit supplementation compared to the control group (Figure 3B). Furthermore, the plasma MDA and T-AOC contents were unaffected by the environment, diet, and the interaction effects (Figures 3C,D). Figure 3E demonstrates that there was a significant interaction between environment and diet on the GSH-Px activity (*P* > 0.05). Compared with the control fed group, birds fed L-Cit had increased GSH-Px activity during HS, but did not differ from TNZ birds fed either control or L-Cit diets.

### L-Cit Supplementation Downregulates Hypothalamic mRNA Expression of Heat Shock Factors (HSF) and Heat Shock Protein (HSP)

**FIGURE 4:**
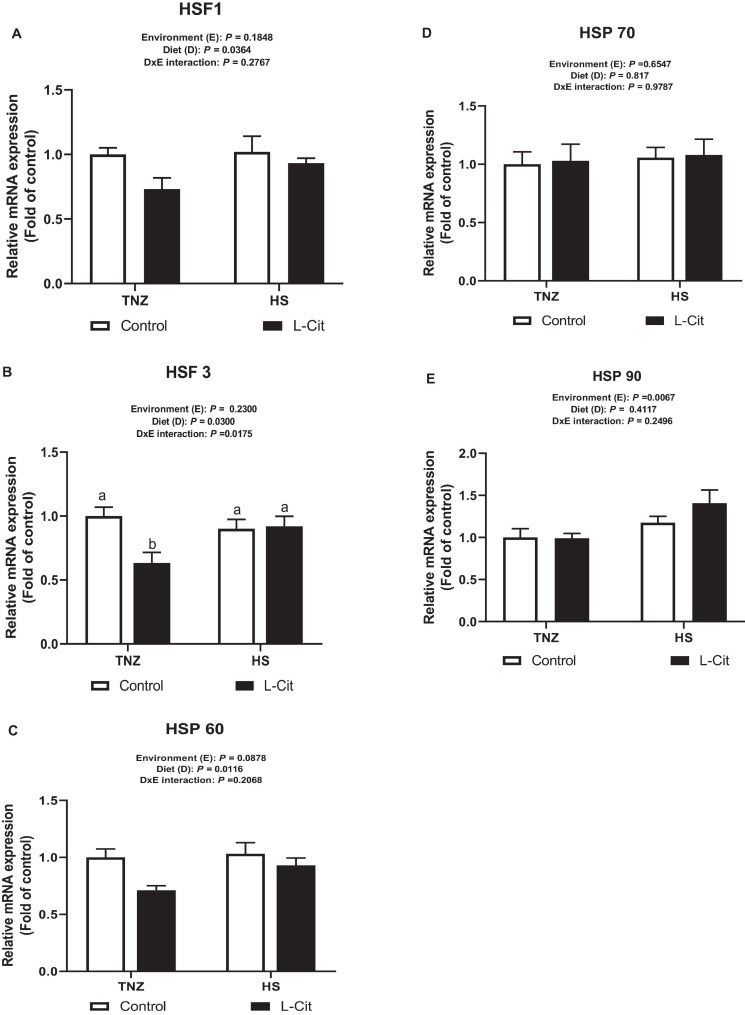
Effect of L-citrulline supplementation on hypothalamic mRNA expression of stress-related genes during acute heat stress. **(A)** Heat shock factor 1 (HSF1). **(B)** Heat shock factor 3 (HSF3). **(C)** Heat shock protein 60 (HSP 60). **(D)** Heat shock protein 70 (HSP 70). **(E)** Heat shock protein 90 (HSP 70). Data are presented as mean ± SEM (*n* = 8). TNZ, thermoneutral; HS, heat stress. ^*a,b*^Different letters indicate significant difference of D×E interaction at *P* < 0.05.

Figure 4A shows that L-Cit supplementation significantly downregulated hypothalamic HSF1 expression compared to control fed group (*P* < 0.05). Similarly, L-Cit supplementation significantly decreased (*P* < 0.05) the mRNA expression of hypothalamic HSF3 compared to the control fed group (*P* < 0.05; Figure 4B). In addition, there was a significant interaction between environment and diet on the HSF 3 expression (*P* < 0.05). It was observed that under TNZ condition, L-Cit supplementation significantly downregulated HSF 3 expression lower than control fed group at TNZ, and the HS groups fed either control or L-Cit diets (*P* < 0.05). Figure 4C shows that L-Cit supplementation significantly downregulated the mRNA expression of HSP 60 compared to the control fed group (*P* < 0.05). HSP 70 expression was not significantly affected (*P* > 0.05) by environment, diet and interaction effects (Figure 4D), however, HS exposure significantly upregulated HSP 90 mRNA expression in the hypothalamus compared to TNZ chickens (Figure 4E).

### NO Concentration and NOS Activity Is Altered by Acute HS and L-Cit Supplementation

**FIGURE 5:**
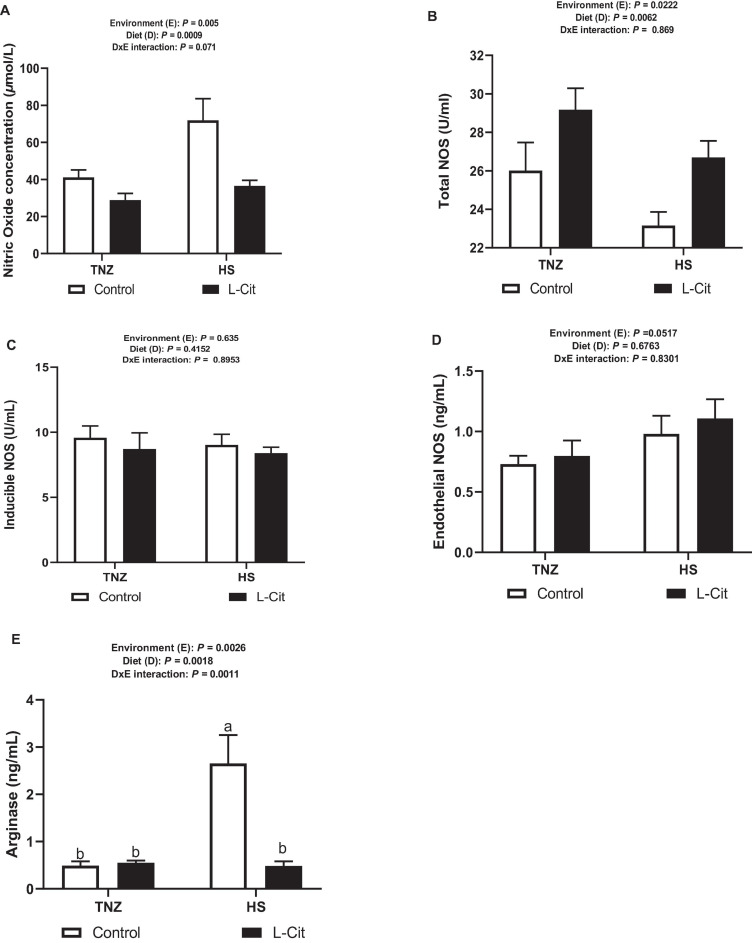
Effect of heat stress and L-citrulline supplementation on nitric oxide production during acute heat stress. **(A)** NO concentration. **(B)** Total NO synthase activity. **(C)** Inducible NO synthase activity. **(D)** Endothelial NO synthase activity. **(E)** Arginase activity. Data are presented as mean ± SEM (*n* = 8). ^*a,b*^Different letters indicate significant difference of D×E interaction at *P* < 0.05.

Peripheral NO concentration was significantly (*P* < 0.05) increased during HS compared to TNZ condition (Figure 5A). L-Cit supplementation significantly diminished NO concentration compared to the control diet group (*P* < 0.05). The tNOS activity was observed to respond in a contrasting manner unlike NO concentration following HS and L-Cit treatment (Figure 5B). HS lowered tNOS activity compared to the TNZ condition (*P* < 0.05). More so, it was observed that L-Cit supplementation increased the tNOS activity compared to the control fed group (*P* < 0.05). The enzyme activity for iNOS and eNOS were unchanged (*P* > 0.05) by environment, diet and interaction effects (Figures 5C,D). Furthermore, arginase activity was significantly increased by HS exposure compared to TNZ group (*P* < 0.05; Figure 5E). L-Cit supplementation was observed to decrease the plasma arginase activity compared to control fed group (*P* < 0.05). In addition, there was a significant interaction effect between environment and diet on the arginase activity (*P* < 0.05). Plasma arginase was induced by HS exposure in control fed broilers, compared to L-Cit fed group under HS, and the TNZ groups fed either control or L-Cit diets.

### L-Cit Influences the Hypothalamic Expression of iNOS-COX2-PGE2 Signaling in Broilers

**FIGURE 6:**
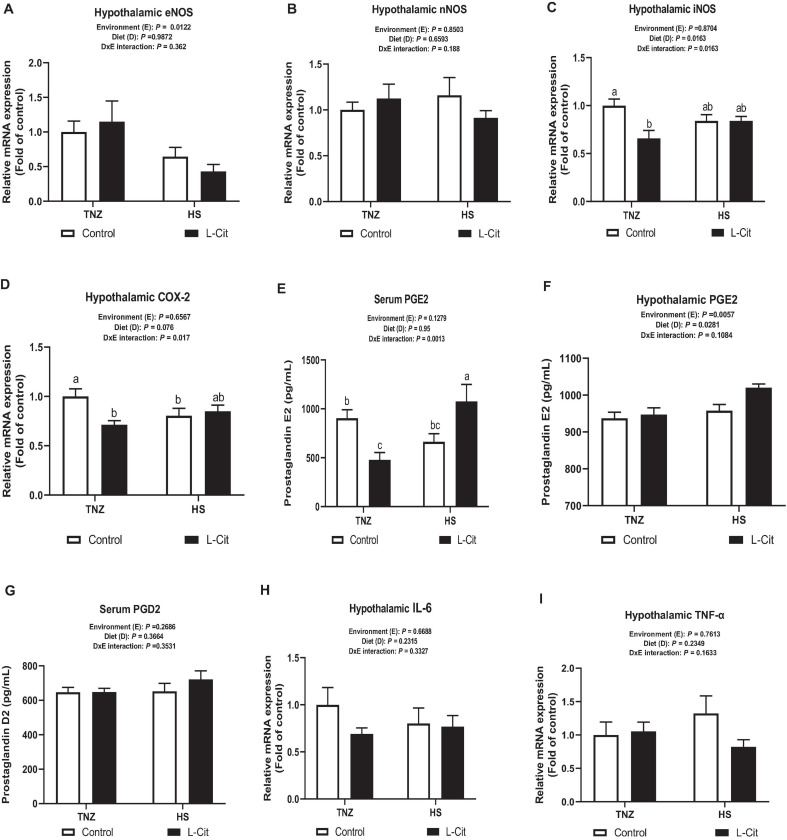
L-Citrulline alters transcriptional expression of hypothalamic NOS2-COX2-PGE2 signaling. **(A)** Endothelial NOS isoform (eNOS). **(B)** Neuronal NOS isoform (nNOS). **(C)** Inducible NOS isoform (iNOS). **(D)** Cyclooxygenase 2 (COX-2). **(E)** Serum Prostaglandin E2 (PGE2). **(F)** Hypothalamic Prostaglandin E (PGE2). **(G)** Serum Prostaglandin D2 (PGD2). **(H)** Interleukin (IL)-6. **(I)** Tumor necrosis factor (TNF)-α. Data are presented as mean ± SEM (*n* = 8). TNZ, thermoneutral; HS, heat stress. ^a,b,c^Different letters indicate significant difference of D×E interaction at *P* < 0.05.

The mRNA expression of hypothalamic NOS isoforms showed that HS significantly downregulated eNOS mRNA expression compared to TNZ group (*P* < 0.05; Figure 6A), whereas, nNOS mRNA expression was not significantly affected (*P* > 0.05; Figure 6B). The hypothalamic mRNA expression for iNOS was decreased by L-Cit supplementation compared to the control fed group (*P* < 0.05; Figure 6C). There was a significant interaction between environment and diet on the hypothalamic iNOS expression (*P* < 0.05). It was observed that under TNZ condition, L-Cit supplementation significantly downregulated iNOS expression lower than the control fed group at TNZ, but did not differ from HS groups fed either control or L-Cit diets. Figure 6D shows that the hypothalamic COX-2 mRNA expression was affected by a significant interaction between environment and diet (*P* < 0.05). It was observed that COX 2 expression was downregulated in L-Cit fed group compared to the control fed group under TNZ condition, but did not differ from the HS groups fed control or L-Cit diets.

The serum PGE2 content was influenced by a significant interaction (*P* < 0.05) between environment and diet (Figure 6E). The L-Cit fed group at TNZ had lowered PGE2 concentration than the control fed group at TNZ, and L-Cit fed group under HS, which had the highest PGE2 content (Figure 6E). The hypothalamic PGE2 concentration was increased during HS condition compared to the TNZ group (*P* < 0.05). It was also observed that L-Cit supplementation increased PGE2 concentration higher than control fed group (*P* < 0.05; Figure 6F). Serum PGD2 concentration was not affected by environment, diet or interaction effect (Figure 6G). Likewise, the mRNA expression of pyrogenic cytokines, IL-6 and TNF-α were unchanged in the hypothalamus (Figures 6H,I).

## Discussion

The present study was designed to gain an understanding of the acute HS paradigm, and how dietary intervention with L-Cit would mediate acute HS effects in broiler chickens. Similar to previous reports, exposure of broilers to acute HS affected thermotolerance by initiating severe hyperthermia and tachypnea. Body temperature measurements including ear temperature, RT and CBT were elevated by HS during the study. Interestingly, L-Cit reduced the RT and CBT intermittently at certain time points during HS and at TNZ. Also, the mean CBT of broilers was lowered by L-Cit supplementation. Reduction in CBT can occur as a beneficial stress response to a variety of stimuli, including, hypercapnia, hypoxia, and hypoglycemia because it lowers oxygen consumption, and energy requirements, thus improving survival (Almeida and Branco, 2001). Although the molecular mechanisms behind this determinant are yet to be elucidated, it is understood that L-Cit may act on the central control of body temperature to achieve hypothermia, but whether this effect is temperature dependent or independent is yet to be fully ascertained. Plasma levels of T_3_ can be considered as a reliable indicator of HS in chickens due to its involvement in regulating heat production, and metabolic rate to achieve thermotolerance (Yahav et al., 1996). As a physiological adjustment to HS, reduction in T_3_ concentration allows for a lowered rate of heat production, metabolism, and energy requirement in chickens (Abu-Dieyeh, 2006). However, few studies report on variations to T_3_ responses during HS (Scheele et al., 1992; Melesse et al., 2011). From our findings, the elevation in serum T_3_ concentration indicates an increased thyroid function and metabolic activity during short-term heat exposure. Increased peripheral conversion of T_4_ to T_3_ may be necessary to meet the increased demand for heat production which is necessary to achieve hyperthermia during HS.

Stress-induced elevation in corticosterone levels is associated with higher resistance and adjustment to the stress stimuli (Quinteiro-Filho et al., 2010; Xu et al., 2018). However, reports on the hypothalamic-pituitary-adrenal axis activation for the release of corticosterone during HS (Zaboli et al., 2019) are inconsistent. Our study revealed that serum corticosterone levels were unchanged during HS and L-Cit supplementation. This finding is similar to previous reports (Lin et al., 2006; Song et al., 2012; Xie et al., 2015). The inconsistent responses reported on corticosterone release have been attributed to variability in the duration of HS, the intensity of thermal stimuli, pulsatile hormonal activity, species of animal, as well as, tissue-specific responses (Dou et al., 2019; Bai et al., 2020). Furthermore, plasma TP, UA, and TCHO in HS broilers did not differ from control birds, demonstrating minimum alterations in nutrient digestion and absorption. In this study, exposure to acute HS challenge suppressed plasma TG and GLU levels, indicating possible perturbations in lipid and carbohydrate metabolism. Typically, heat challenge elevates plasma GLU levels to permit stress resistance and survival during the “fight or flight” response (Xie et al., 2015). In addition, Lin et al. (2006) had reported that acute HS did not influence glucose levels in broilers, whereas circulating levels of glucose in broilers were diminished during chronic HS. Our findings revealed that L-Cit increased plasma GLU levels but lowered the TG content at TNZ. In line with previous reports, L-Cit had been shown to elicit beneficial effects on plasma TG, and was also implicated in the regulation of glucose and lipid metabolism (Jegatheesan et al., 2015).

Heat stress is closely associated with oxidative stress due to an imbalance between ROS production and the antioxidants defense system (Roushdy et al., 2018). L-Arginine metabolism to L-Cit by NOS allows for the formation of NO, and when in excess can result in oxidative damage, mitochondrial dysfunctions, and lipid peroxidation (Mishra and Jha, 2019). From our study, HS modulated SOD activity, two-fold higher than TNZ, but the effect of L-Cit was evident via increased CAT activity. Controversial reports exist on the influence of HS on antioxidant enzyme activity (Madkour et al., 2020), however in a recent review it has been explained that exposure to high ambient temperature allows for a compensatory increase in the antioxidant enzymes activity such as SOD, GSH-Px, and CAT, in order to afford short term protection against excessive free radicals produced (Goel et al., 2021). Under HS condition, L-Cit increased GSH-Px action to counteract ROS formation, although the plasma MDA and T-AOC contents were not influenced. Previously, L-Cit had been reported to possess potent antioxidant effects via its ability to scavenge hydroxyl radicals and inhibit ROS formation (Coles, 2007). Also, it had been shown that dietary L-Cit improved the antioxidant status via increased SOD, CAT, and T-AOC, but decreased the MDA content in laying hens (Uyanga et al., 2020). Thus, L-Cit stimulation of antioxidant responses may serve to combat HS-induced oxidative damage to certain extents. In addition, stress-induced lymphoid organ involution is a common index for evaluating the immunosuppressive index of stress (Kimura et al., 1996). In this study, acute HS did not affect the spleen index, rather, L-Cit lowered the spleen index, which may be indicative of an altered immune response, such as lymphocyte depletion (Elmore, 2006). Nevertheless, further investigations are needed to understand the influence of L-Cit on lymphoid organ development.

Stress induction leads to the release of HSPs which are critical for thermoprotection, repair of denatured proteins, protein folding, and prevention of subsequent stress effects (Calderwood et al., 2017). The induction of heat shock response during stress condition is primarily anchored on the activation and binding of the HSFs and heat shock elements, to allow for the transcription of HSPs (Creagh et al., 2000; Aramburu et al., 2014). HSF1 and 3 are the two major HSFs responding to heat shock in avian species, thus they are considered as avian-specific HSFs (Xie et al., 2014). Interestingly, our results showed that L-Cit lowered HSF1 expression, which may have resulted in HSP 60 downregulation. More so, L-Cit downregulated HSF3 expression in the hypothalamus under thermoneutral condition. Essentially, during HS, the rapid response of HSFs is necessary for immediate transcription of HSPs, as genes necessary for cell survival are activated, whereas, less essential genes are downregulated (Xie et al., 2014). Since HSPs are constitutively expressed within living organisms, these findings suggest that L-Cit may act to preserve the activity of heat response metabolites under thermoneutrality. Also, it was found that HS induced HSP90 expression, probably to function in protecting the structural and metabolic integrity of tissues against stress-induced injury (Hao and Gu, 2014; Madkour et al., 2021). Furthermore, Zhao et al. (2016) had revealed that there exists a strong positive relationship between NO and HSPs expression (*r* ≥ 0.9), such that NO had stronger interactions with HSP60 and HSP90, compared to other HSPs. These findings give additional insights into the cross-talk that exists between HSPs and NO-mediated signaling.

Dietary factors can function to modulate the availability or deficiency of NO, rendering important implications for health and disease (Luiking et al., 2010). Previously, L-Cit supplementation was shown to increase the serum-free arginine, citrulline, and ornithine levels, as well as nitric oxide synthesis in laying hens (Uyanga et al., 2020). In this present study, it was revealed that HS significantly modulated circulating NO concentration and arginase activity but inhibited tNOS activity. Alongside this, L-Cit supplementation elicited contrasting effect by decreasing NO concentration and arginase activity but increased tNOS activity. A clear understanding of the opposing action of these two factors with respect to NO generation is yet to be unraveled and necessitates further studies. From existing literature, it is understood that the utilization of L-Cit as an alternative to restore L-arginine homeostasis and NO production may be regulated by feedback mechanisms to limit NO overproduction (Cynober et al., 2010, 2013). Therefore, an increased L-arginine availability arising from L-Cit supplementation may in turn acts as a sensor to prevent potentially neurotoxic levels of NO in the blood circulation (Wu et al., 2009; Cynober et al., 2010). This report is in line with our findings of L-Cit-induced reduction in NO concentration. Additionally, the intracellular NOS activity is reflected by tNOS, which comprises both the constitutive and iNOS isoforms (Uyanga et al., 2020). However, findings from this study revealed that L-Cit-induction of tNOS activity did not correspond with an increase in NO production, whereas, L-Cit was able to lower arginase activity. L-Arginine can be catabolized either by NOS to NO and L-Cit or it is hydrolyzed by arginase to ornithine and urea (Rath et al., 2014). As such, there are multiple cross-inhibitory interactions between these two arginine metabolic pathways which compete for the common substrate, L-arginine (Kocyigit et al., 2004; Rath et al., 2014). It is understood that when excess L-arginine is supplied in the diet, the kidney arginase activity is increased resulting in the rapid degradation of L-arginine to urea and ornithine (via the urea cycle), but where this degradation proceeds too rapidly, L-arginine availability to serve in NO production may be impaired (Ruiz-Feria et al., 2001). L-Cit-induced arginase inhibition had been reported as a protective mechanism against impairment in endothelium-dependent relaxation (El-Bassossy et al., 2013). More so, arginase inhibition restores L-arginine availability for NOS action since both enzymes actively compete for L-arginine (Cynober et al., 2010). Thus, these results demonstrate the regulatory role of L-Cit via arginase inhibition to allow for increased L-arginine availability, tNOS activity and subsequent NO production. Noteworthy is the fact that the L-Cit-induced tNOS activity in this study did not translate for higher NO production. Therefore, we propose that since the control and L-Cit based diets were formulated with equal L-arginine levels, L-arginine availability would have probably contributed to impeding the efficiency of NO regeneration *in vivo*.

The iNOS isoform is generally stimulated by inflammatory stimuli and contributes to NO production under stressful conditions (Yu et al., 2018). This study revealed that under thermoneutrality, L-Cit supplementation downregulated the central expression of iNOS, and elicited a similar pattern to the COX2-PGE2 expression. Investigation into the iNOS-COX2-PGE2 signaling showed that the hypothalamic expressions of iNOS and COX2 were downregulated by L-Cit under thermoneutral condition, although, L-Cit differentially influenced serum PGE2 at TNZ and HS temperatures. L-Cit initiated an inhibitory role on peripheral PGE2 levels at thermoneutrality but stimulated PGE2 release during HS, which corresponded with the increased hypothalamic PGE2 levels during HS exposure. PGE2 can increase body temperature via coordinated increment in heat production and a concomitant decrease in heat loss (Milton, 1998). Furthermore, the role of PGE2 has been widely investigated in the central control of body temperature regulation (Ayoub et al., 2004), and it has been described as the principal mediating factor for the generation of hyperthermia during fever induction (Osaka, 2010; Conti, 2016). Therefore, PGE2 induction or reduction at different temperatures validates its involvement in body temperature regulation. From our results, it is evident that the decrease in PGE2 levels under thermoneutral condition was mediated by iNOS-dependent downregulation of COX-2 signaling. More so, the L-Cit-suppression of body temperature may be attributed to a modified thermoregulatory set point resulting from the altered activity of thermosensitive neurons in the POA, which is induced by locally released PGE2 (Almeida et al., 2004). However, the unresponsiveness of pro-inflammatory cytokines such as interleukin (IL)-6, and tumor necrosis factor (TNF)-α, would suggest the interplay of other immunomodulatory molecules in activating the iNOS-COX2-PGE2 signaling.

## Conclusion

Altogether, findings from this study reveal that acute HS exposure significantly elevated body temperature and altered the redox status, heat shock response, and nitric oxide synthesis in broilers. L-Cit supplementation acted to lower the body temperature and HSFs activation, improved antioxidant enzyme responses, and diminished circulating NO levels. Also, it was evident that inflammatory mediators of the iNOS-COX2-PGE2 signaling have a role to play in the regulation of body temperature at thermoneutrality and during HS conditions. Undoubtedly, these findings provide the knowledge base for further studies to ascertain the role of temperature and nitric oxide in L-Cit’s actions.

## Data Availability Statement

The raw data supporting the conclusions of this article will be made available by the authors, without undue reservation.

## Ethics Statement

The animal study was reviewed and approved by Institutional Animal Care and Use Committee at the College of Animal Science and Veterinary Medicine of Shandong Agricultural University.

## Author Contributions

VU and HL designed the research. VU, MW, and TT performed the animal experiments and data collection. VU analyzed, interpreted, and presented the data. HJ, JZ, XW, and OO contributed in the research design and results interpretations. All authors read, reviewed and approved the final manuscript.

## Conflict of Interest

The authors declare that the research was conducted in the absence of any commercial or financial relationships that could be construed as a potential conflict of interest.

## Publisher’s Note

All claims expressed in this article are solely those of the authors and do not necessarily represent those of their affiliated organizations, or those of the publisher, the editors and the reviewers. Any product that may be evaluated in this article, or claim that may be made by its manufacturer, is not guaranteed or endorsed by the publisher.
